# Red Ginseng Treatment for Two Weeks Promotes Fat Metabolism during Exercise in Mice

**DOI:** 10.3390/nu6051874

**Published:** 2014-05-05

**Authors:** Hyejung Hwang, Jisu Kim, Jonghoon Park, Heayeon Yun, Woo-Kwang Cheon, Bokyung Kim, Chi-Ho Lee, Heajung Suh, Kiwon Lim

**Affiliations:** 1 Laboratory of Exercise Nutrition, Department of Physical Education, Konkuk University, Seoul 120, Neungdong-ro, Gwangin-gu, Seoul 143-701, Korea; E-Mails: hfilm@konkuk.ac.kr (H.H); kimpro@konkuk.ac.kr (J.K.); jonghoonp@konkuk.ac.kr (J.P.); yunhy@konkuk.ac.kr (H.Y.); hjsuh21@hanmail.net (H.S.); 2 Department of Physical Education, Keimyung University, Daegu 704-701, Korea; E-Mail: wk11106@kmu.ac.kr; 3 Department of Medicine, Konkuk University, Chungju 380-701, Korea; E-Mail: bkkim2@kku.ac.kr; 4 Department of Food Science and Biotechnology of Animal Resources, Konkuk University, Seoul 143-701, Korea; E-Mail: leech@konkuk.ac.kr

**Keywords:** red ginseng, endurance training, carbohydrate oxidation, fat oxidation, glycogen

## Abstract

PURPOSE: Red ginseng (RG) has been reported to improve the blood and organ lipid profile when combined with exercise. However, the effect of RG on energy metabolism during exercise is poorly understood. Therefore, this study was designed to investigate whether RG treatment alters fat utilization during exercise; METHODS: We used seven-week-old ICR mice (*n* = 42). RG (1 g/kg) was administered orally daily during two weeks of endurance training. All mice were randomized into two groups: training only group (CON group) and training with RG group (RG group). Endurance training consisted of 20~25 m/min on a slope of 8° for one hour five times a week. After a two-week experimental period, we measured substrate utilization during exercise at the same intensity and duration of training using a respiratory calorimetry chamber. Mice were dissected for glycogen measurement of muscles and liver before, immediately after, and one hour after the exercise; RESULT: Fat oxidation during the initial 20 min of the one-hour exercise significantly increased in the RG group compared to the CON group. In addition, the liver glycogen stores significantly decreased immediately after the one-hour exercise compared to at rest in the RG group, but did not differ between immediately after the one-hour exercise and at rest in the RG group. The glycogen concentration in white and red gastrocnemius muscle did not differ between the groups immediately after the one-hour exercise; CONCLUSIONS: These results suggest that RG treatment for two weeks promotes fat oxidation and a glycogen-sparing effect during exercise. This might lead to a delay in peripheral fatigue during endurance exercise performance.

## 1. Introduction

The energy needed for exercise is not only stored in the body, but it is also derived from dietary carbohydrate, protein, and fat that is broken down or oxidized through their respective metabolic pathways [1]. To increase endurance exercise capacity, it is important to utilize fat metabolism effectively and/or have a glycogen-sparing effect in the liver and muscle during exercise of the peripheral tissues [2,3,4]. Peripheral fatigue is mainly caused by depletion of stored energy in peripheral tissues and it is thought to reduce lactate levels in the muscle. Thus, to increase the ability to efficiently exercise it is necessary (1) to increase stored glycogen in the liver and muscle; (2) promote oxidation of stored fat; and (3) promote blood circulation.

Ginseng, as a natural remedy, improves psychologic function, exercise performance, and immune function. Many animal and human studies showed that intake of ginseng has beneficial effects. It enhances phagocytosis, natural killer cell activity, and production of interferon; improves physical and mental performance; it increases resistance to stress; and affects hypoglycemic activity [5]. These beneficial effects are attributed to ginsenosides (Rg1, Rb1, Rg2) and saponin. In particular, ginsenosides has been shown to make up a larger proportion of “red ginseng”. Red ginseng is produced by steaming and drying ginseng. During this heat process, ginsenosides undergo chemical changes resulting in a compound with the potential to induce special physiologic activities [6,7,8].

Many studies reported on the anti-fatigue effect of ginseng extract or red ginseng. A 10-day *in vivo* administration of ginseng extract modulates peroxisome proliferator-activated receptor-α function, which regulates the expression of a number of genes critical for lipid and lipoprotein metabolism, and significantly increases serum concentrations of total cholesterol, triglycerides, and high-density lipoprotein cholesterol [9]. In a study by Song *et al.* [10], mice were fed a high-fat diet containing RG for 13 weeks and the authors found that genes associated with lipid metabolism were down-regulated; these genes are usually up-regulated by a high-fat diet. In studies demonstrating the effect of ginseng on exercise performance, acute ginseng intake after a three-hour swimming exercise improved recovery from fatigue, suggesting an effect of ginseng on endurance exercise performance. Additionally, mice treated with acute ginseng extract had higher blood glucose and lower plasma free fatty acid (FFA) levels after swimming for 30 min [11,12].

Most previous studies analyzed the effect of ginseng extract or red ginseng on blood lipid profiles before and after exercise, but did not determine the alteration of energy metabolism during exercise, such as respiratory quotient level or fat oxidation. Therefore, this study was designed to investigate whether RG administration influences the alteration of fat utilization during exercise using a respiratory calorimetric chamber and measuring glycogen levels in liver and muscles. The results of the present study will provide further insight into the beneficial effect of RG on efficient metabolic regulation during exercise.

## 2. Materials and Methods

### 2.1. Animals and Treatment

Seven-week-old male ICR mice (*n* = 42) were used. The mice were purchased from Orient Bio Inc. (Seongnam, Korea). All mice were housed in standard plastic cages under controlled conditions of humidity (50%) and temperature (23 ± 1 °C) with alternating 12-h cycles of light (08:00 to 20:00) and darkness (20:00 to 08:00). Mice were allowed to adapt to the laboratory housing for 1 week. Forty-two male mice were divided into 2 groups: control (CON, *n* = 21) and RG administered (RG, *n* = 21). They were given free access to water and a non-purified commercial diet (5L79, Orient Bio Inc., Seongnam, Korea), containing (g/kg) crude protein, 180; crude fat, 52; crude fiber, 52; minerals, 57; and carbohydrate, 368. The protein, fat, and carbohydrate ratio (%) based on calories was 21:14:65, and gross and metabolizable calorie contents of the diet were 4.04 and 3.21 kcal/g, respectively. Body weight and food intake of each mouse were measured (Innotem IB-6100 balance, company, city, Korea). All experimental procedures were carried out at the Animal Experiment Research Center of Konkuk University. This study was conducted in accordance with the ethical guidelines of the Konkuk University Institutional Animal Care and Use Committee.

**Table 1:** Ginsenosides composition in Red-Ginseng Extract (mg/g).

Ingredient	Content
Rg1	0.71
Re	0.93
Rf	1.21
Rf1	0.78
Rg2(s)	1.92
Rg2^®^	1.29
Rb1	4.62
Rc	2.41
Rb2	1.83
Rd	0.89
Rg3(s)	2.14
Rg3^®^	0.91
**Total**	**19.64**

### 2.2. Red Ginseng Extract

RG extract dissolved in distilled water was administered to the RG group daily at a dose of 1 g/kg body weight (included 19.64 mg ginsenosides/g RG extract) for 2 weeks [13]. The CON group was treated with vehicle only (distilled water 5 mL/kg body weight). RG was obtained from Korea Ginseng Corp. (Seoul, Korea). Details on the levels of ginsenosides in RG are shown in Table 1. The mice in the control group received the same amount of distilled water.

### 2.3. Training Methods

We used aerobic running exercise protocol with minor modification [14]. After 3 days of an adaption period for the treadmill, all mice were forced to run at 20 m/min (running speed) and was raised by maintaining the slope at 8° for 50 min once a day, 5 times a week, on the first week of training. The running time per a day was increased by 25 m/min and maintained the slope at 8° on the last week of training (65%–70% of 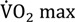). 

### 2.4. Measurement of Metabolic Rates

Resting metabolic rate was measured 1 week before and 2 weeks after the exercise period as previously described [14]. After the 2-week experimental period, we measured substrate utilization during exercise at the same intensity and duration of training method using a respiratory calorimetry chamber. Two hours before the experiment, mice were put in a metabolic chamber to reduce stress. They were given free access to water and food. Flow velocity within the chamber was set to 1.2 L/min and measured for 24 h.

### 2.5. Gas Analysis

Respiratory gas was measured using an open-circuit apparatus based on previous studies [15,16]. The O_2_ uptake and CO_2_ production were measured using a mass analyzer (model RL-600, Alco System, Chiba, Japan; used a gas analyzers) and a switching system (model ANI6-A-S, Alco System). All flow rates were kept constant at 3 L/min. O_2_ uptake and CO_2_ production were used to calculate the respiratory exchange ratio, carbohydrate oxidation, and fat oxidation in the mice.

### 2.6. Blood Analysis

Blood samples were collected before, immediately after, and 1 hour after the exercise. Plasma was prepared by centrifugation. Plasma glucose was measured using commercial kits (Asan Pharmaceutical Co., Hwaseong-si Gyeonggi-do, Korea), the plasma FFA level using a non-esterified fatty acid kit (Wako Pure Chemical Industries), and the plasma insulin level was determined with an enzyme-linked immunosorbent assay kit (Morinaga Bioscience Laboratory, Yokohama, Japan).

### 2.7. Glycogen Analysis

Mice were dissected for the glycogen measurement in muscles and liver before, immediately after, and 1 h after the exercise. Glycogen was measured in perchloric acid extract using the amyloglucosidase method [17]. Approximately 30 mg of tissues were sampled on ice. We added 0.5 mL of 2N HCL and incubated the samples for 2 h at 96 °C before adding 1.5 mL of 0.67 M NaOH. After centrifugation at 5000 g and 4 °C for 5 min, glycogen hydrolyzed to glucose as the supernatant. A 100 µL sample of glucose was taken and 1 mL of reaction buffer (containing 1M Tris-HCL (pH 8.0), 100 mM MgCl_2_, 200 mM adenine triphosphate, 500 nM dithiothreitol, 10 mM nicotinamide adenine dinucleotide phosphate, hexokinase, and glucose-6-phosphate dehydrogenase) was added and incubated for 30 min at 37 °C. Then, the sample was put on ice to stop the reaction. The glucose content was analyzed in 96-well plates using a spectrophotometer at a wavelength of 340 nm.

### 2.8. Statistical Analysis

The SPSS 19.0 program was used for the statistical analysis of this study (SPSS, Inc., Chicago, IL, USA). The effect of time and group was analyzed using two-way repeated measures analysis of variance. Since a significant interaction was not detected, a *post hoc* Tukey test was used for multiple comparisons in the CON groups and the RG groups. Differences were considered significant at *p* < 0.05.

## 3. Results

### 3.1. Body Weight and Food Intake

Changes in body weight and food intake during the two-week experimental period are shown in Table 2. The initial body weight, final body weight, body weight gain, total amount of food intake, and daily food intake was not different between the two groups.

**Table 2:** The change of body weight, food intake.

	CON	RG
Initial body weight (g)	35.1 ± 1.6	34.7 ± 1.4
Final body weight (g)	36.9 ± 2.0	36.1 ± 1.2
Body weight gain (g)	1.6 ± 1.2	1.4 ± 1.0
Total amount of food intake (g/2weeks)	73.0 ± 6.8	77.6 ± 5.9
Daily food intake (g/day)	4.5 ± 0.4	4.8 ± 0.3

Values are presented as means ± standard error; CON: no treatment with training; RG: RG treatment with training.

### 3.2. Carbohydrate Oxidation and Fat Oxidation during Exercise

**Figure 1:**
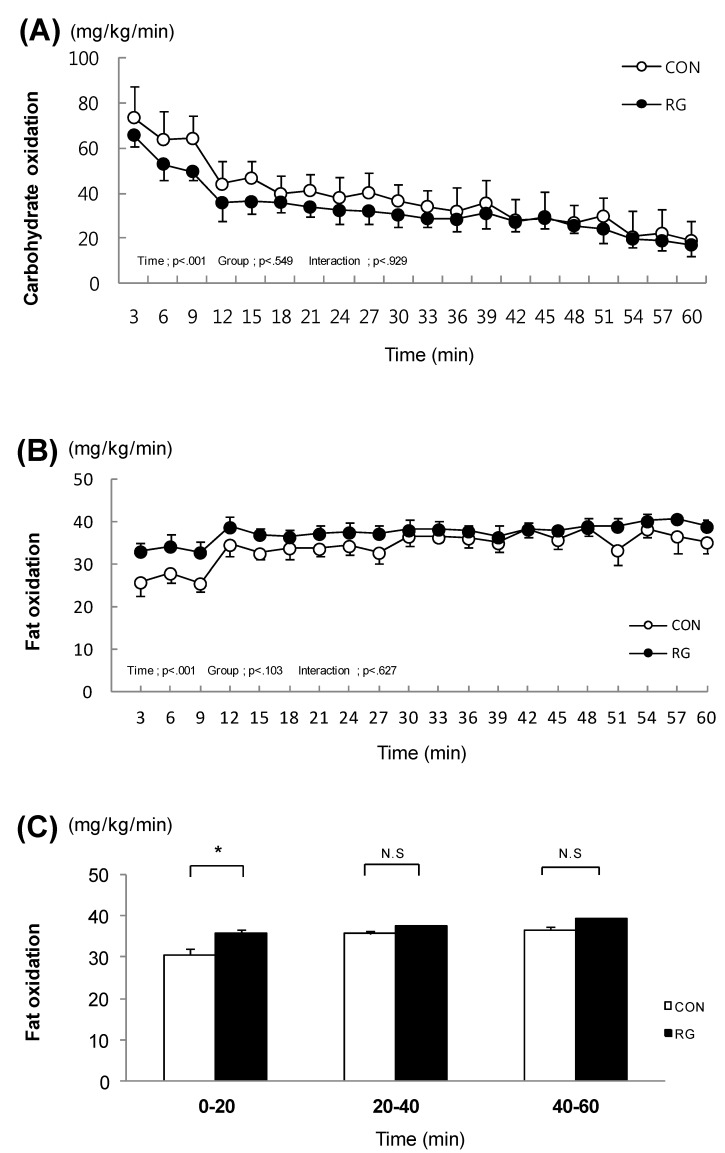
The change in carbohydrate oxidation and fat oxidation during exercise for 1 h. (**A**). The change in carbohydrate oxidation during exercise for 1 h; (**B**). The change in fat oxidation during exercise for 1 h; (**C**). Fat oxidation at every 20 min. Values are presented as means ± standard error (*n* = 21). * *vs.* CON *p* < 0.05; CON: no treatment with training, RG: RG treatment with training.

The exercise lasted for one hour at an intensity of about 65%–75% 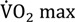. After the two-week experimental period, carbohydrate oxidation during exercise was not different between the two groups (Figure 1A). In addition, after the experimental period, fat oxidation during exercise was not different between the two groups (Figure 1B). In contrast, when examining each session of exercise separately, fat oxidation during the initial 20 min of the one-hour exercise was significantly higher in the RG group (35.5 ± 0.8 mg/kg/min) than in the CON group (30.5 ± 1.5 mg/kg/min) (Figure 1C).

### 3.3. Plasma Parameters

**Figure 2:**
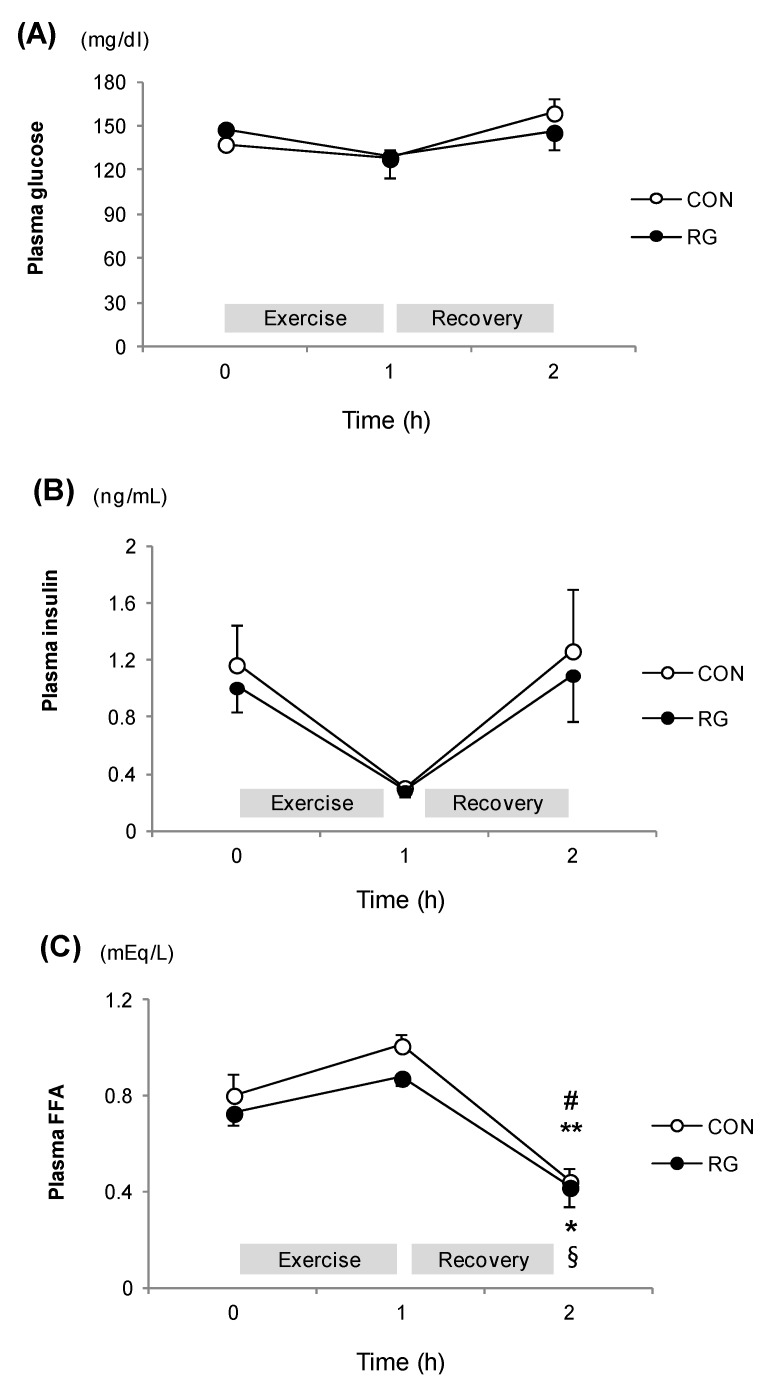
The change in plasma parameters during exercise and after exercise for one hour. (**A**): Glucose; (**B**): Insulin; (**C**): Free fatty acid level at rest, after exercise, and recovery in the CON and RG groups. Values are presented as means ± standard error (*n* = 14). # *vs.* Rest in RG *p* < 0.05; ** *vs.* After exercise in RG, *p* < 0.05; * *vs.* Rest in CON, *p* < 0.01; § *vs.* After exercise in CON, *p* < 0.01; CON: no treatment with training; RG: RG treatment with training.

The plasma concentrations of glucose and insulin did not differ between the groups at any time point (Figure 2A,B). The plasma concentrations of FFA decreased after recovery from the exercise for 1 h in both groups, but it did not differ between the groups at any time point (Figure 2C).

**Figure 3:**
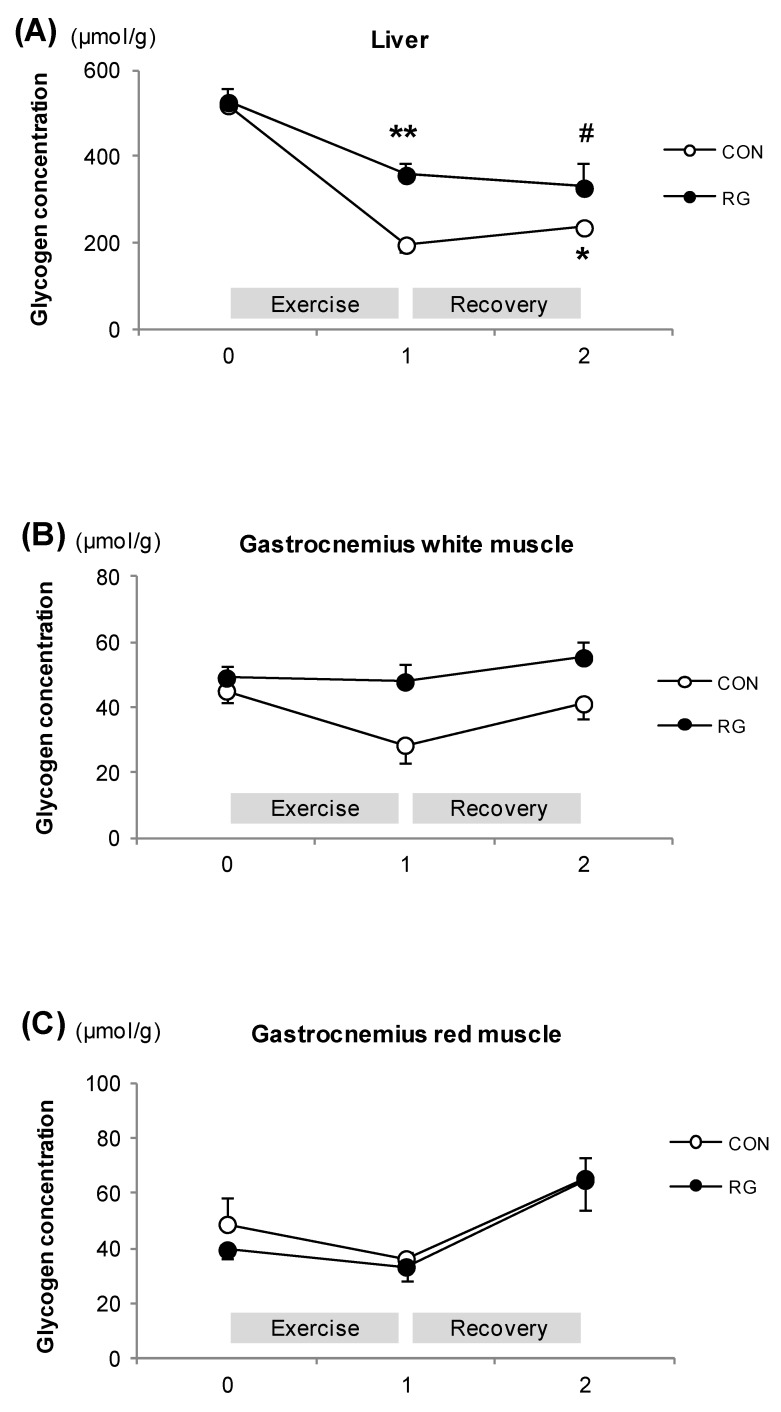
The change in glycogen concentrations during exercise and after exercise for one hour. (**A**): Liver; (**B**): Gastrocnemius-white muscle; (**C**): Gastrocnemius-red muscle at rest, after exercise, and recovery in the CON and RG groups. Values are represented as means ± standard error (*n* = 14). # *vs.* Rest in RG, *p* < 0.01; ** *vs.* Rest in RG, *p* < 0.01; * *vs.* Rest in CON, *p* < 0.05 (two-way analysis of variance group effect ***); CON: no treatment with training; RG: RG treatment with training.

### 3.4. Glycogen Concentrations

In the CON group, the liver glycogen stores significantly decreased after recovery from the exercise for one hour compared to when at rest. However, this decrease was not observed in the RG group (Figure 3A). The glycogen concentrations in white gastrocnemius muscle tended to be higher in exercise-trained mice treated with RG at the immediately after the one hour exercise (*p* = 0.069) and those in and red gastrocnemius muscle did not differ between the groups at any time point (Figure 3B,C).

## 4. Discussion

The major findings of the present study were the following: firstly, fat oxidation during the initial 20 min of a one-hour exercise period was significantly higher in exercise-trained mice that received RG compared to mice that were only exercise-trained; secondly, the liver glycogen stores significantly decreased immediately after the one-hour exercise period in the mice that were only exercise-trained, but this decrease was not observed in exercise-trained mice that received RG.

Several studies suggested that RG intake reduced body weight and fat content [18,19,20]. RG intake down-regulated genes associated with lipid metabolism, which are up-regulated by a high-fat diet [10,11]. Based on these previous studies, we expected that RG intake would further increase fat oxidation when combined with endurance training, which is known to increase the rate of fatty acid mobilization in intramuscular fat [21]. In the present study RG intake increased fat oxidation after the initial 20 min of the one-hour exercise period in exercise-trained mice. As one of reasons for the increase in fat oxidation at the only initial phase, we cautiously assume that the effect of RG intake on the fat oxidation might have been diluted by the exercise training. Not only intake of red ginseng, but moderate intensity of exercise increases fatty acid availability during exercise. We also observed a glycogen-sparing effect after the one-hour exercise period in exercise-trained mice that received RG. This glycogen-sparing effect in the liver after the one-hour exercise period was also seen in white gastrocnemius in exercise-trained mice treated with RG, though it did not reach statistical significance. Taken together, these results indicate that administration of RG for two weeks in combination with endurance training might have a synergistic effect on increased fat oxidation during exercise.

We found higher glycogen levels after one-hour exercise in exercise-trained mice that with RG. Tang *et al.* [22] demonstrated that glycogen levels in the liver were higher after a prolonged swimming exercise in mice that received 20(*R*)-ginsenoside Rg3 for two weeks. Although this study used a different type of exercise and a different level of intensity than our study, it suggests that ginsenosides might influence hepatic glycogen metabolism. In addition, rats that received acute ginseng extract had higher plasma glucose and lower FFA levels after a 30-minute swimming exercise compared to control rats [11]. However, our study did not show different levels in plasma biomarkers between groups despite the higher hepatic glycogen levels in exercise-trained mice treated with RG. It is considered that plasma glucose and FFA levels might have changed during the initial phase of the one-hour exercise. We observed higher fat oxidation during the initial phase and thereby hepatic glycogen level increased after one hour of exercise in order to supply glucose to the muscle.

Xiong *et al.* [23] demonstrated that ginsenoside Rb1 administration promoted fat oxidation and had a glycogen-sparing effect. Especially, they showed that the food intake in the Rb1 treatment group was lower than in the control group, probably via appetite inhibition. In contrast, the effect of appetite inhibition by red Ginseng treatment was not observed in the present study and this may be due to that the difference of the dosage and experimental duration between Xiong *et al.*’study [23] and our study. The dosage of Rb1 administered was 10.0 mg/kg body weight and four weeks of duration in Xiong *et al.*’s study, but 4.6 mg/kg body weight and two weeks in this study.

Twelve weeks of intake of Korea Red Ginseng enhanced the insulin signaling pathway by increasing phosphorylation of IR-b, IRS-1, Akt and GSK3a/b and by increasing the glucose transporter isoform (GLUT4) translocation in skeletal muscle [20]. Exercise training is the most potent stimulus to increase skeletal muscle GLUT4 expression [24]. We showed that glycogen concentrations in white gastrocnemius muscle tended to be higher in exercise-trained mice treated with RG, but it did not reach statistically significant level. Particularly, it was unclear why glycogen concentration in liver was higher after exercise in RG group compared with CON group, although this result was not observed in gastrocnemius muscles. Thus, it will be necessary for us to examine the effect of RG on glucose metabolism related to gene expression in liver and muscles in a future study.

The present study had several limitations. Firstly, we did not perform a run-time to exhaustion test, which can provide more information on the beneficial effects of the intervention on endurance performance. Secondly, we didn’t set the exercise and food control so that could not discuss the effect of RG intake or exercise training on parameters related to energy metabolism. To clarify the synergistic effects of RG administration in combination with exercise on energy metabolism in more detail, it would be important to add a resting group to the present experimental setting. However, we clearly revealed that RG intake could enhance fat oxidation during exercise in exercise-trained mice as this result was not observed in exercise-trained mice that did not with RG.

## 5. Conclusions

In conclusion, RG intake for two weeks promotes fat oxidation and the glycogen-sparing effect during exercise. Therefore, consuming RG when training can improve endurance exercise performance.
